# Early detection of anxiety symptoms in Autism Spectrum Disorder: An exploratory study in a Spanish sample of 3–6 year old children

**DOI:** 10.1371/journal.pone.0318408

**Published:** 2025-01-31

**Authors:** Montserrat Durán-Bouza, Silvia Gómez-Ríos, Margarita Cañadas-Pérez, Juan-Carlos Brenlla-Blanco

**Affiliations:** 1 Psychology Department, University of A Coruña, A Coruña, Spain; 2 Occupational Sciences Department, Catholic University of Valencia, Valencia, Spain; Azienda Ospedaliera Universitaria Policlinico Umberto I Rome, ITALY

## Abstract

Current research often overlooks anxiety symptoms in preschoolers with Autism Spectrum Disorder (ASD), focusing on older children. This study examines anxiety symptomatology primarily in young children with ASD by analyzing data from 82 Spanish children aged 3- to 6 years. Parents completed the Anxiety Scale for Children with ASD and the Social Communication Questionnaire. Results indicate that 30.48% of the children exhibit significant anxiety symptoms, “with anxious uncertainty” being the most prevalent. Factor analysis reveals three principal components of the Anxiety Scale, supporting its reliability. Regression analysis highlights stereotypical behaviors as significant predictors of anxiety levels. These findings underscore the importance of early assessment and adaptation of assessment tools for preschoolers with ASD. Stereotypical behaviors are identified as key predictors of anxiety in this population.

## Introduction

Research consistently highlights that anxiety disorders are a prevalent and disabling challenge for individuals with Autism Spectrum Disorder (ASD) [1]. Children with ASD are estimated to have a significantly heightened risk of anxiety, with rates of approximately 40%, compared to their typically developing peers [2].

While previous studies have primarily focused on identifying anxiety disorders in ASD aged six and older, recent evidence suggests the presence of early signs of anxiety symptoms in younger children with ASD. Consequently, recent research efforts have intensified, aiming to detect key anxiety symptoms at an early age [3–7].

Prevalence data for anxiety symptoms in young children show significant variability, influenced by factors such as sample type, age, IQ, assessment tools, and information sources. For example, clinical samples, using the Child Behavior Checklist (CBCL) report prevalence rates ranging from 16.7% to 31.8%, while the Early Childhood Interview-4 (ECI-4) estimates a prevalence of 62% [7, 8]. Community samples show a broader range, with rates varying from 1.6% to 38% [6, 9, 10].

In summary, research findings on the overall prevalence of anxiety in young children with ASD show marked inconsistencies, with reported values ranging from 1.6% to 62% [11].

Studies comparing the severity of anxiety between children with ASD and typically developing children or those with other neurodevelopmental disorders yield mixed results, depending on the instruments used [12, 13]. The relationship between cognitive development and anxiety in ASD remains inconclusive, with conflicting findings reported in the literature [7, 8, 14].

The clinical manifestations of anxiety in ASD lack consensus, further complicated by the intricate interplay between anxiety symptoms and the core characteristics of the disorder [15]. Some studies suggest that anxiety may present as a comorbid disorder, as defined by the diagnostic criteria in the Diagnostic and Statistical Manual of Mental Disorders, Fifth Edition (DSM-5) [16]. Conversely, other studies argue that children with ASD exhibit atypical anxiety symptoms that reflect heightened and clinically harmful anxiety linked to disorder-specific characteristics. These symptoms, include fears of change, novelty, and uncommon phobic stimuli, such as those associated with sensory sensitivities (e.g., fears of loud noises) [17–19].

Discrepancies in the anxiety symptoms across studies highlight the need for standardized approaches. Table 1 summarizes data from frequently used assessment instruments, shedding light on common anxiety symptoms and disorders in ASD. Most of these instruments are used as screening tools.

**Table 1:** Most frequent anxiety symptoms in ASD in young children according to the assessment instrument used.

Study	Anxiety measure	Age (range)	*Most frequent anxiety disorders or/and symptoms*	Less frequent anxiety symptoms/and symptoms
Gadow [20]	ECI-4	3–5 years	Specific phobia Social phobia Compulsions	
Weisbrot [21]	ECI-4	3–5 years	Compulsions Specific phobia Social anxiety Generalized anxiety symptoms	
Hayashida [22]	ECI-4 CBCL	3–6 years	Generalized anxiety Separation Anxiety	
Sukholdsky [23]	ECI-4	3–7 years	Withdraws in uncomfortable social Sleep problems	
Llanes [6]	CBCL	4-7years	Too fearful Too dependent Fears of animals, situations, or places	Gets upset when separated from parents
Mayes [24]	CADS	1–16 years	Fears of toilets Fears of objects Mechanical things	
Cervantes and Matson [25]	BISCUIT-2	18–36 mos.	Fears of people Fear of animals	Specific situations
Keen [5]	ASC-ASD	5–6 years	Uncertainty fear Generalized fear	Safety of parents Physical symptoms
Bischof [26]	PAS-R	mean = 4.7 years	Specific phobias Generalized phobias	
Driscoll [27]	KSADS-E PARS SC/PAS	3–7 years	Generalized anxiety Specific phobia	

While most research focuses on school-age children, general anxiety noted to be more prevalent in older children, whereas symptoms of separation anxiety are more common in younger age groups [2]. An autism-specific anxiety measure (ASC-ASD) highlights “anxious uncertainty” as a frequent symptom in school-aged children with ASD [5, 28–31].

Anxiety symptoms in children with ASD tend to worsen with age, negatively impacting academic, social, and adaptive functioning [14, 32–37]. However, current research often relies on instruments designed for neurotypical children, potentially limiting both accuracy and consistency. The ASC-ASD scale has emerged as a promising tool for assessing anxiety symptoms in young children with ASD [30].

In this research, we utilized the ASC-ASD scale to gather insights from parents of young children diagnosed with Autism Spectrum Disorder (ASD), focusing on prevalent anxiety symptoms during these developmental stages. The main objectives of this study were as follows:

1. To outline the prevalent anxiety symptoms frequently observed in ASD during early childhood.
2. To assess the efficacy of the ASC-ASD scale in identifying anxiety symptoms among children with ASD aged 3 to 6 years.
3. To explore potential correlations between anxiety symptoms and key clinical characteristics of Autism Spectrum Disorder (ASD), as assessed by the Social Communication Questionnaire (SCQ).

## Method

### Participants

The sample consisted of 82 children (aged 3- to 6 years) with ASD from various autonomous communities in Spain. Parents or legal caregivers of children who had received a diagnosis of Autism Spectrum Disorder (ASD) were invited to participate voluntarily through different associations of families affected by this condition.

The inclusion criteria specified that participants should be between 3 and 6 years old and have a clinical diagnosis of ASD, Asperger’s syndrome, or Pervasive Developmental Disorder (PDD) confirmed by a qualified professional (medical doctor, psychologist, or psychiatrist).

The mean age of the participants was 4.6 years (range 3–6 years). Among them, 29 (35.4%) were female and 53 (64.6%) were male. In most cases, mothers (70 participants compared to 12 fathers) completed the questionnaires, with gathered information on the children’s demographic characteristics, as well as details regarding the disorder and anxiety symptoms. Table 2 summarizes the data related to associated disorders.

**Table 2:** Sociodemographic characteristics of participants.

	*n*	%
Age		
3	23	28.0
4	13	15.9
5	21	25.6
6	25	30.5
Gender		
Female	30	36,6
Male	52	63,4
Associated disorders		
Attention deficit hyperactivity disorder	5	7.6
Attention deficit hyperactivity disorder + Eating Disorder	5	7.6
Intellectual development disorder plus Eating Disorder	3	4.5
Sleep Disorders	3	4.5
Eating disorders	7	10.6
Food intolerance	2	3.0
More than two comorbid disorders	5	7.6
Others	15	22.7
No additional diagnosis	21	31.8
Education		
Specific classroom	28	34.1
General education school without a specific classroom and without support	11	13.4
Attended a regular general education school with support outside the classroom	6	7.3
Did not attend school	37	45.2

### Measures

A Sociodemographic Questionnaire was developed to collect information about the data providers, including the children’s age, gender, additional diagnoses, educational settings, and other relevant details.

To assess parents’ self-reported observations of their child’s anxiety symptoms, the Anxiety Scale for Children with Autism Spectrum Disorder (ASC-ASD) by Rodgers [30] was used. Available in both parent-report and self-report versions, this dimensional measure is specifically designed to evaluate common anxiety manifestations in children and adolescents with ASD. Adapted from the Revised Children’s Anxiety and Depression Scale (RCADS) by Chorpita [38], the ASC-ASD addresses three specific aspects of anxiety in autism: sensory hypersensitivity, intolerance to uncertainty, and the presence of specific phobias.

Parents or caregivers rated items on a 4-point scale (0 to 3), with a maximum possible score of 72. Scores ≥20 indicate "significant anxiety symptoms" while scores >24 suggest a "more specific indication of significant anxiety." The 24 items are grouped into four subscales: Separation Anxiety (maximum score: 15); Uncertainty (maximum score: 24), Performance Anxiety (maximum score: 15), and Anxious Arousal (maximum score: 18).

The ASC-ASD scale demonstrates excellent psychometric properties, with a Cronbach’s alpha coefficient of .94 for the full scale, and subscales reliabilities .89 for Performance Anxiety, .87 for Separation Anxiety, .91 for Anxious Uncertainty, and .87 for Anxious Arousal [30]. Its convergent validity is supported by a correlation of .91 with the Screen for Anxiety and Related Emotional Disorders (SCARED) [39], while its discriminant validity is indicated by a .65 correlation with the Child Depression Inventory (CDI-2) [40]. Although originally designed for children aged 8- to 16 years, it has been successfully applied to children as young as 5- to 6 years old [5].

The Social Communication Questionnaire (SCQ) by Rutter [41] was also employed. This tool consists of 40 items completed by parents based on their observations of social interaction difficulties, communication challenges, and stereotyped behaviors. While it is typically used with children aged 4 years and older, research suggests its potential applicability to children aged 2 to 4 years [42]. Responses are dichotomous (Yes/No), scored as 1 or 0, and summed to produce a total score that reflects atypical behaviors in the social, communicative, and restricted/repetitive behavior domains. Internal consistency values for the SCQ range from .84 to .93 [41].

### Procedure

The study was conducted following approval from the Ethics Committee for Research and Teaching of the University of Coruña, (file number 2020/0021).

Initially, a digital information document, was prepared, outlining the study’s characteristics and including a confidentiality statement and an informed consent form. This document aimed to encourage voluntary participation from parents or legal guardians of children aged 3 to 6 with Autism Spectrum Disorder (ASD). A digital questionnaire, accessible via a link, was created to collect data, incorporating the Sociodemographic Questionnaire, the ASC-ASD scale, and the SCQ. Outreach efforts included collaboration with ASD organizations, educational centres, and professional associations in Spain, as well promotion through social media platforms and webinars.

Contact with participating families was facilitated through professional organizations and associations related to ASD form Spain. These entities distributed an email containing three distinct links:

1. Detailed Study Information: This include the study’s objectives, assurances of confidentiality and a request for informed consent.
2. Sociodemographic Questionnaire: This link directed families to provide relevant demographic and background information.
3. ASC-ASD and SCQ Scales: This link allowed participants to complete the anxiety and social communication assessment.

Data collection was carried out exclusively these online to minimize participant fatigue and reduce the burden of information processing. Families who chose to participate were required to provide written informed consent. A total of 85 parents participated in the study, with one exclusion due to incomplete data and two exclusions for children older than 7 years. Completing the questionnaire took approximately 15 minutes. Data collection was conducted between June 21, 2021, and December 1, 2023.

### Data analyses

Data were analyzed using the SPSS statistical software package version 28. Descriptive analyses were performed to summarize the results of the ASC-ASD scale and the SCQ questionnaire.

Given that the ASC-ASD scale was originally validates for children aged 6 years and older, an exploratory factor analysis was conducted to evaluate the scale’s validity for younger age groups. Additionally, stepwise regression analyses were performed to determine the extent to which SCQ scores could explain variability in the ASC-ASD scores.

## Results

### Descriptive analysis of anxiety symptoms using the ASC-ASD scale

The descriptive analysis of the scores obtained on the ASC-ASD scale by the 82 participants revealed a mean total score of 16.72 (SD = 11.51). Scores ranged from a minimum of 1 to a maximum of 53. Among the participants, 7 scored between 20 and 24, indicating anxious symptomatology, while 18 scored above 24, signifying significant anxiety. Consequently, 30.48% of the sample exhibited significant anxious symptomatology.

Table 3 presents the mean scores, standard deviations and range of scores for each subscale. The Uncertainty subscale had the highest scores, with participants achieving up to 22 points out of a possible 24. This was followed by the Performance Anxiety subscale (13 points out of a possible 15), the Separation Anxiety subscale (12 points out of a possible 15), and the Anxious Arousal subscale (11 points out of a possible 18).

**Table 3:** ASC-ASD mean scores subscale.

Subscale	Minimum	Maximum	Mean	SD
Separation Anxiety	0	12	4.88	3.12
Uncertainty	0	22	7.38	5.79
Performance Anxiety	0	13	2.02	2.92
Anxious Arousal	0	11	2.07	2.27

As previously mentioned, the Uncertainty subscale had the highest total and mean scores per item. Item 16(“My child always needs to be prepared before things happen”) received the highest score. In this subscale, only two parents reported an absence of symptoms.

Within the Performance Anxiety subscale, Item 7 (“My child worries about doing badly at schoolwors”) was rated as "often”or “always" by 18% of parents. Conversely, the items with the lowest scores were Item 2 ("My child worries about what other people think of him/her, e.g., that he/she is different"), which 79.26% of parents rated as “never” and Item 17 ("My child feels afraid that he/she will make a fool of him/herself in front of people"), which 76.82% of parents also rated as”never”.

In the Separation Anxiety subscale, Item 18 ("My child worries about being away from me") had the highest score, with almost half of the parents (41.46%) perceiving this behavior as occurring “always” or “almost always”. Item 11 ("My child worries when in bed at night because he /she does not like to be away from his/her parents or family") was scored as “always” or “usually” by 29.26% of parents.

The items within the Anxious Arousal subscale generally received the lowest scores. The highest-scoring item, Item 1 ("My child suddenly gets a scared feeling when there is nothing to be afraid"), was rated as “frequently occurring” by 12.19% of parents. The items with the lowest scores were Item 22 ("My child suddenly becomes dizzy or faint without reason "), which 93.90% of parents rated as “never”, and Item 8 (“My child feels so anxious that he/she cannot breathe, for no reason"), which 90.24% of parents rated as “never”.

### Exploratory factor analysis of the ASC-ASD scale for anxiety in children aged 3–6

To evaluate the utility of the ASC-ASD scale for assessing anxiety in children aged 3- to 6 years, an exploratory factor analysis was conducted. Bartlett’s test of sphericity was statistically significant (*χ*^*2*^ (276) = 1112.04, *p* ≤ .001), and the Kaiser-Meyer-Olkin (KMO) measure of sampling adequacy was 0.08, exceeding the recommended threshold of 0.32, and indicating the suitability of the data for factor analysis.

A principal component analysis identified five principal components with eigenvalues greater than 1. Factor 1 accounted for 35.11% of the variance, Factor 2 for 12.90%, Factor 3 for 7.23%, Factor 4 for 5.07%, Factor 5 for 4.69%. Combined, these factors explained 65% of the total variance. Table 4 details the items included in each factor.

**Table 4:** Factors obtained through the principal components method and items in each factor.

Factor	Items	Total
1	1, 9, 11, 16, 18, 20, 21, 23	8
2	2, 4, 7, 15, 17	5
3	3, 5, 6, 10, 12	5
4	8,13, 14, 22	42
5	19, 24	

The internal consistency of the items included in each factor was calculated using Cronbach’s alpha coefficient. The results indicated high reliability indices for all five factors (*α* = 0.86, 0.86, 0.80, 0.71 and 0.73, respectively). The overall reliability of the scale for the sample used in this study was 0.91.

Among the eight items included in Factor 1, three corresponded to the “Uncertainty", subscale, three to the "Separation Anxiety" subscale, and two to the "Anxious Arousal" subscale. The five items in Factor 2 were exclusively form the "Performance Anxiety" subscale. Factor 3 comprised five items, three form the "Uncertainty" subscale and two "Anxious Arousal" subscale. Of the four items Factor 4, three belonged to the "Anxious Arousal" subscale, and one to the "Uncertainty" subscale. Finally, Factor 5 consisted of two items from the "Separation Anxiety" subscale.

### Prevalence of ASD characteristic assessed by the SCQ

Regarding the results of the Social Communication Questionnaire (SCQ), 70 out of 82 children (85.4%) obtained scores indicative of difficulties across the three areas assessed: social interaction, communication difficulties, and stereotyped behaviors. The mean total score was 17.28 (range: 5–31; *SD* = 6.29), with a cut-off score of 11 for identifying difficulties in early childhood.

Table 5 summarizes the mean scores, standard deviation and range of scores obtained by participants in each of the areas. The Social Interaction Difficulties domain included 15 of the 40 total items, while the Communication Difficulties domain contained 13 items, and the Stereotyped Behaviors domain consisted of eight items.

**Table 5:** Mean scores obtained in the areas assessed by the SCQ.

Areas	Minimum	Maximum	Mean	SD
Difficulties in social interaction	0	14	5.76	3.69
Communication difficulties	0	9	5.27	2.25
Stereotyped behaviors	0	8	5.13	1.83

### Regression analysis of SCQ scores in explaining ASC-ASD scores variability

Stepwise regression analyses were conducted to evaluate the extent to which SCQ scores could explain the variability in ASC-ASD scores. First, a stepwise regression analysis was performed using the total score on the ASC-ASD scale as the dependent variable and the three SCQ-assessed areas–social interaction, communication difficulties, and stereotypic behaviors–as independent variables.

The regression equation was statistically significant (*F*(1,80) = 13.45, *p*≤.001) with an *R*^*2*^ value of .14. This indicates that the regression model explained 14% of the variability in the total ASC-ASD scores, with stereotypic behaviors emerging as the key predictor. The regression equation was: 4.52 + 2.37 *(stereotypic behaviors. This implies that the total ASC-ASD scores increases by 2.37 points for every unit increase in the SCQ score for stereotypic behaviors.

For the Separation Anxiety subscale, the regression equation also statistically significant (*F*(1,80) = 6.41, *p* = .013)with an *R*^*2*^ value of .07. This model explained 7% of the variability in Separation Anxiety scores based solely on stereotypic behaviors. The regression equation was: 2.50 + 0.46 *(stereotypic behaviors). Thus, scores on the Separation Anxiety subscale increase by 0.46 points for each unit increase in the stereotypic behaviors score.

In the case of Anxious Uncertainty, stepwise regression analysis yielded two statistically significant models. The first model included stereotypic behaviors (*F*(1,80) = 14.40, *p*≤ .001, with a *R*^*2*^ value of .15, explaining 15% of the variability in scores on this subscale. Uncertainty can be explained by the regression model that only includes stereotypic behaviors. However, the second model includes, in addition to stereotypic behaviors, communication difficulties (*F*(2,79) = 9.75, *p*≤ .001), increasing the *R*^*2*^ value to .20, which was a change in *R*^*2*^ of .05. The resulting regression equation was: -1.11 + 1.16 *(stereotypic behaviors) + 0.55 *(communication difficulties). Therefore, scores on the Uncertainty subscale would increase by 1.16 points with stereotypic behaviors and 0.55 points with communication difficulties.

For Performance Anxiety, two statistically significant models were identified. The first model included social interaction difficulties as the sole predictor (*F*(1,80) = 9.58, p = .003), with a *R*^*2*^ value of .11. This indicates that 11% of the variability in Performance Anxiety subscale scores could be explained by social interaction difficulties alone.

The second model incorporated stereotypic behaviors alongside social interaction difficulties as predictors (F(2,79) = 7.61, *p* = .001), resulting in an R^2^ value of .16—an increase of .05 compared to the firs model. The regression equation was: 3.51–0.28 *(social interaction difficulties) + 0.37 *(stereotypic behaviors). This suggest that Performance Anxiety scores decrease by 0.28 points with increasing social interaction difficulties, but increase by 0.37 points with higher stereotypic behaviors scores.

Lastly, the stepwise regression analysis for the Anxious Arousal subscale identified a single statistically significant model (*F*(1,80) = 8.76, *p* = .004). The *R*^*2*^ value was: .10, indicating that 10% of the variability in Anxious Arousal subscale scores could be explained by stereotypic behaviors. The regression equation obtained was: 0.08 + 0.39 *(stereotypic behaviors), where the total scores on the Anxious Arousal subscale increase 0.39 points from the scores on the SCQ score for stereotypic behaviors.

## Discussion and conclusions

### Anxiety levels in young children with ASD: The role of “Uncertainty”

This study, examined anxiety levels in young children with Autism Spectrum Disorder (ASD) using the ASC-ASD scale. The findings reveal that a substantial proportion of the sample (30.48%) exhibited significant anxiety symptoms, demonstrating consistent manifestation of anxiety across diverse cohorts of autistic children.

However, the mean total score (*M* = 16.72) derived from parental responses on the ASC-ASD scale was notably lower than those reported in studies involving older children. For instance, Rodgers [30] documented a mean score of 26.19 for children aged 8–15 years, while de Den Houting [28, 29] found mean scores of 27.4 and 26.68 in children aged 9–12 years. Keen [5], in a study focused on 5-6-year-olds, reported a mean score of 22.82. These results are consistent with the hypothesis that anxiety symptoms in children with ASD may intensify with age [14, 31, 33], underlining the importance of early intervention to address anxiety and prevent its exacerbation over time.

Among the ASC-ASD subscales, the "Uncertainty" subscale yielded the highest scores. This suggest that anxiety in young children with ASD is closely linked to difficulties in coping with novel or stressful situations and sensory overload. These findings align with previous research, indicating that intolerance to uncertainty plays a central role in the development of anxiety in autistic children [30, 31, 43].

Intolerance to uncertainty is critical increasingly recognized as a dispositional risk factor for anxiety. This underscores the necessity of interventions targeting this construct to alleviate anxiety symptoms in children with ASD [44, 45]. One notable intervention is the "Coping with Uncertainty in Everyday Situations (CUES)" program developed by Rodgers [46], which focuses on improving tolerance to uncertain situations in children aged 6 to 16 years. Expanding such interventions to younger children could significantly enhance their quality of life, as well as that of their families. Early implementation of strategies to manage uncertainty may reduce anxiety levels and foster greater emotional resilience in young children with ASD.

### ASC-ASD scale utility for early assessment

The exploratory factor analysis conducted in this study demonstrates good psychometric properties of the ASC-ASD scale for assessing anxiety in children aged 3 to 6 years. However, findings suggest that adapting the original scale to include four or five dimensions may enhance its applicability for this younger age group. These results partially align with the findings of Beneytez-Barroso [43], who reported a variation in the number of items within each factor in their Spanish adaptation of the scale for children aged 6 years and older. Despite these differences, the overall factor structure remains consistent with the original four-factor configuration, as also highlighted by Beneytez-Barroso [43].

Moreover, the study reveals a potential relationship between Uncertainty and Separation Anxiety, which could stem from young children with ASD seeking parental support to navigate unfamiliar or challenging situations. As Keen [5], observed, children with autism may be particularly susceptible to Separation Anxiety due to the critical role of parental support in understanding and addressing their unique needs. Consequently, children rely on their parents to manage uncertain circumstances. Future research should delve deeper into this relationship, paving the way for interventions aimed at reducing dependency on parents and alleviating associated Separation Anxiety.

The study also indicates that items associated with Performance Anxiety appear to form a distinct dimension (Factor 2), incorporating two items from the Separation Anxiety subscale, which are consolidated into a another dimension (Factor 5). Interestingly, approximately half of the items from the Anxious Uncertainty subscale are distributed across Factors 3 and 4, along with the majority of items from the Anxious Arousal subscale.

This distribution underscores a deeper connection between physiological anxiety symptoms and the need for predictability and alignment with cognitive schemas, which may be particularly influenced by sensory hypersensitivity. Meanwhile eight items converge into Factor 1, encompassing aspects of anxious uncertainty, Separation Anxiety, and Anxious Arousal. These findings highlight the multifaceted nature of anxiety symptoms in young children with ASD and underscore the need for refined tools to capture these nuances effectively.

### Interplay of restricted behaviors and anxiety in children with ASD: Insights

The results obtained from the SCQ; data clearly demonstrate that a significant proportion of the participating children displayed elevated scores across all three areas assessed by the questionnaire. Regression analyses, further elucidated the predictive role of these areas in explaining the variability of anxiety levels, as assessed by the ASC-ASD scale.

A key finding highlights the importance of stereotypical behaviors in predicting both overall anxiety levels and those specific to individual subscales. A clear correlation emerges between rigid–core characteristics of Autism Spectrum Disorder (ASD)–and the intensity of anxiety symptoms within this population.

These findings are consistent with prior research, which has frequently linked elevated anxiety levels in children with ASD to the presence of repetitive behaviors [23, 47]. Longitudinal studies have consistently shown that the severity of restricted and repetitive behaviors (RRBs) serves as a predictive factor for subsequent anxiety, suggesting a predominantly unidirectional relationship between the two constructs [32]. This insight underscores the potential for clinicians to use patterns of stereotypical behaviors as an early marker for anxiety, facilitating timely intervention to mitigate long-term psychological distress.

Despite the robust evidence connecting Restricted and Repetitive Behaviors (RRBs) with anxiety, it remains unclear whether this relationship is uniform across all RRB subtypes or specific to certain categories. Current research provides strong evidence linking insistence on sameness and restricted interests with anxiety. However, the association between anxiety and other RRB subtypes, such as sensory behaviors or repetitive motor movements, remains ambiguous. Conflicting findings may stem from varying definitions of RRBs; some studies classify these behaviors into distinct subtypes, while others group them into broader domains such as low-order and high-order RRBs [48].

Further support for these findings comes from a longitudinal study by Baribeau [49], which revealed that children displaying behavioral rigidity, such as a strict adherence to routines during preschool years, were more likely to experience both heightened insistence on sameness and elevated anxiety levels between the ages of 8 and 11. These results provide valuable insight into the complex relationship between specific RRB subtypes and anxiety, highlighting the importance of early behavioral patterns predictors of future emotional challenges.

While the results of this study are promising they also emphasize the need for further research. Validating the ASC-ASD scale in larger, more diverse populations, and using confirmatory factor analyses alongside convergent validity studies, will strengthen its applicability. Integrating parent reported data with observations from additional sources and utilizing diverse assessment methods will provide a more comprehensive understanding of anxiety in children with ASD.

Finally, it is important to acknowledge the difficulties parents face in distinguishing anxiety symptoms within the context of ASD. These challenges highlight the necessity of developing multiple reliable methods to accurately identify and characterize anxiety in this population. Such efforts are crucial for ensuring early detection and effective intervention strategies.

### Conclusions

1. This study reveals a notable prevalence of anxiety symptoms among young children with Autism Spectrum Disorder (ASD), with approximately 30.48% of the sample displaying significant symptoms. These findings suggest a consistent manifestation of anxiety across diverse groups of autistic children.
2. The results indicate that mean anxiety scores, as measured by the ASC-ASD scale, were lower in younger children compared to older cohorts. This aligns with previous research suggesting that anxiety symptoms in children with ASD may intensify with age, emphasizing the importance of early interventions to address anxiety and mitigate its progression.
3. The "Uncertainty" subscale yielded the highest scores, reflecting heightened anxiety in response to novel or stressful situations and sensory overload. Intolerance to uncertainty is identified as a significant dispositional risk factor, highlighting the necessity for interventions specifically targeting this construct to reduce anxiety symptoms in ASD.
4. The study underscores the predictive value of stereotypical behaviors in determining both overall anxiety levels and subscale-specific scores. This finding underscores the association between rigid, repetitive behaviors intrinsic to ASD and the severity of anxiety symptoms within this population.
5. While the ASC-ASD scale demonstrates considerable promise as a tool for assessing anxiety in young children with ASD, further validation is necessary. Future research should on larger and more diverse samples, employing confirmatory factor analyses and studies of convergent validity. Additionally, integrating multiple reliable assessment methods is essential for accurately identifying and characterizing anxiety in children with ASD.
6. Longitudinal studies are critical for tracking the development and evolution of anxiety predictors in children with ASD. These investigations can enhance our understanding of symptom trajectories over time and inform the design of effective interventions to minimize the long-term impact of anxiety in this population.

Overall, despite limitations such as the relatively small sample size, this study supports the potential utility of the ASC-ASD scale in assessing anxiety in young children with ASD. These findings encourage further research to refine the scale’s factor structure and validate its overall applicability with larger cohorts.
